# Advances in medical treatment of breast cancer in 2022

**DOI:** 10.1002/cai2.46

**Published:** 2023-02-21

**Authors:** Jingtong Zhai, Yun Wu, Fei Ma, Virginia Kaklamani, Binghe Xu

**Affiliations:** ^1^ Department of Medical Oncology National Cancer Center, National Clinical Research Center for Cancer, Cancer Hospital, Chinese Academy of Medical Sciences and Peking Union Medical College Beijing China; ^2^ Division of Hematology, Oncology UT Health San Antonio MD Anderson Cancer Center San Antonio Texas USA

**Keywords:** breast cancer, chemotherapy, targeted therapy, endocrine therapy, immunotherapy

## Abstract

Breast cancer has replaced lung cancer as the most common malignancy worldwide. The 5‐year survival rate of breast cancer has reached 90%. Systemic treatment of breast cancer has developed into a mature system including chemotherapy, targeted therapy, endocrine therapy and immunotherapy. This article summarizes the annual progress of breast cancer chemotherapy, targeted therapy, endocrine therapy and immunotherapy in 2022, providing valuable information for future research to better guide individualized treatment of breast cancer, thereby improving the prognosis and quality of life of breast cancer patients.

## Graphical abstract

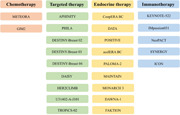

**Abbreviations**
ADC: antibody‐drug conjugates
AI: aromatase inhibitor
ASCO: American Society of Clinical Oncology
CCCA: complete cell cycle arrest
CBR: clinical benefit rate
CDK4/6: cyclindependent kinase 4/6
CI: confidence interval
CPS: combined positive score
DAR: drug‐to‐antibody ratio
DFS: disease‐free survival
EFS: event free survival
ER: estrogen receptor
ESMO: European Society for Medical Oncology
GGI: genome grade index
HER2: human epidermal growth factor receptor 2
HR: hazard ratio
HR: hormone receptor
IARC: International Agency for Research on Cancer
IDFS: invasive disease‐free survival
IHC: immunohistochemistry
ILD: interstitial lung disease
ITT: intention to treat
MBC: metastatic breast cancer
NCCN: National Comprehensive Cancer Network
NSAI: nonsteroidal aromatase inhibitor
OFS: ovarian function suppression
ORR: objective response rate
OS: overall survival
PD‐1: programmed death protein‐1
PD‐L1: programmed death ligand‐1
PFS: progression‐free survival
PgR: progesterone receptor
PI3K/AKT/mTOR: Phosphoinositide 3‐kinase/protein kinase B/mammalian target of rapamycin
PRO: patientreported outcomes
RCB: residual cancer burden
SABCS: San Antonio Breast Cancer Symposium
SERD: selective estrogen receptor degrader
SG: sacituzumab govitecan
SoC: standard of care endocrine therapy
T‐DM1: trastuzumab‐emtansine
T‐DXd: trastuzumab deruxtecan
THPy: pyrotinib plus trastuzumab and docetaxel
TKI: tyrosine kinase inhibitor
TNBC: triple‐negative breast cancer
TPBC: triple‐positive breast cancer
tpCR: total pathologic complete response
Trop‐2: trophoblast cell‐surface Ag‐2
TTF: time to failure
VEX: vinorelbine plus cyclophosphamide and capecitabine

## 1 BACKGROUND

According to the International Agency for Research on Cancer (IARC), breast cancer has replaced lung cancer as the most common malignancy worldwide [1]. The 5‐year survival rate of breast cancer has reached 90% [2], which has improved significantly in the past 20 years due to the molecular classification diagnosis and personalized treatment of breast cancer, as well as the advancement of antineoplastic drugs. Systemic treatment of breast cancer has developed into a mature system including chemotherapy, targeted therapy, endocrine therapy and immunotherapy. In recent years, the application of omics technologies (e.g., genomics, transcriptomics, metabolomics, and proteomics) has increased dramatically, thereby providing new means for individualized treatment of breast cancer patients. This article reviews major advances in the field of breast cancer through 2022 (Table 1) and hopefully provides insight into future prospects.

**Table 1:** Summary of clinical trials.

	Trial name	Phase	Disease status	Treatment	Patient type	References
Chemotherapy	METEORA	2	Palliative	Metronomic oral vinorelbine + cyclophosphamide + capecitabine vs. weekly paclitaxel	ER‐positive, HER2‐negative metastatic breast cancer	[3]
	GIM2	3	Adjuvant	Dose dense chemotherapy vs. standard‐interval schedule chemotherapy	Node‐positive breast cancer	[4]
Targeted therapy	MUKDEN01	2	Neoadjuvant	Pyrotinib + letrozole + dalpiciclib	HR‐positive, HER2‐positive breast cancer	[5]
	APHINITY	3	Adjuvant	Pertuzumab + trastuzumab + chemotherapy vs. placebo + trastuzumab + chemotherapy	Early HER2‐positive breast cancer	[6]
	ATEMPT	2	Adjuvant	T‐DM1 vs. paclitaxel + trastuzumab	Stage I HER2‐positive breast cancer	[7]
	PHILA	3	Palliative	Pyrotinib + trastuzumab + docetaxel vs. placebo + trastuzumab + docetaxel	HER2‐positive metastatic breast cancer	[8]
	DESTINY‐breast 02	3	Palliative	T‐DXd vs. physician's choice chemotherapy	HER2‐positive metastatic breast cancer	[9]
	DESTINY‐breast 03	3	Palliative	T‐DXd vs. T‐DM1	HER2‐positive metastatic breast cancer	[10]
	DESTINY‐breast 04	3	Palliative	T‐DXd vs. investigator's choice chemotherapy	HER2‐low metastatic breast cancer	[11]
	DESTINY‐breast 01	1	Palliative	T‐DXd	HER2‐positive breast cancer with stable brain metastases	[12]
	TUXEDO‐1	2	Palliative	T‐DXd	HER2‐positive breast cancer with active brain metastases	[13]
	DAISY	2	Palliative	T‐DXd	HER2‐low breast cancer with brain metastases	[14]
	HER2CLIMB	3	Palliative	Tucatinib + trastuzumab + capecitabine vs. trastuzumab and capecitabine	HER2‐positive breast cancer with brain metastases	[15]
	U31402‐A‐J101	1/2	Palliative	HER3‐DXd	HER3‐expressing metastatic breast cancer	[16]
	TROPiCS‐02	3	Palliative	Sacituzumab govitecan vs. physician's choice of chemotherapy	HR‐positive, HER2‐negative metastatic breast cancer	[17]
Endocrine therapy	CoopERA BC	2	Neoadjuvant	Giredestrant + palbociclib vs. anastrozole + palbociclib	ER‐positive, HER2‐negative, postmenopausal, untreated early breast cancer	[18]
	ADAPT and ADAPT cycle	3	Neoadjuvant	Endocrine therapy vs. chemotherapy	HR‐positive, HER2‐negative, intermediate to high‐risk early breast cancer	[18]
	ASTRRA	3	Adjuvant	OFS + tamoxifen vs. tamoxifen	ER‐positive, premenopausal, ≤45 years, early breast cancer	[19]
	DATA	3	Adjuvant (extended)	3 years of anastrozole vs. 6 years of anastrozole	HR‐positive, postmenopausal, disease‐free after 2–3 years of adjuvant tamoxifen	[20]
	Unicancer ASTER 70s	3	Adjuvant	Endocrine therapy + chemotherapy vs. endocrine therapy	ER‐positive, HER2‐negative, ≥70 years	[21]
	POSITIVE		Adjuvant	Stop endocrine therapy, get pregnant, childbirth, resume endocrine therapy	HR‐positive，premenopausal，after 18–30 months adjuvant endocrine therapy	[22]
	ELAINE 1	2	Palliative	Lasofoxifene versus fulvestrant	ER‐positive, HER2‐negative, locally advanced/metastatic breast cancer, ESR1 mutation, after progression on AI and CDK4/6 inhibitor	[23]
	acelERA BC	2	Palliative	Giredestrant versus physician choice of endocrine monotherapy	ER‐positive, HER2‐negative, locally advanced/metastatic breast cancer	[24]
	EMERALD	3	Palliative	Elacestrant versus standard of care endocrine therapy	ER‐positive, HER2‐negative, metastatic breast cancer, progression on prior endocrine and CDK4/6 inhibitor therapy	[25]
	PALOMA‐2	3	Palliative	Palbociclib + letrozole versus placebo + letrozole	ER‐positive, HER2‐negative, advanced breast cancer, as first‐line treatment	[26]
	FUTURE	2	Palliative	Fulvestrant + palbociclib	HR‐positive, HER2‐negative, advanced/metastatic breast cancer, progression to fulvestrant monotherapy	[27]
	MONALEESA		Palliative	Ribociclib + endocrine therapy vs. placebo + endocrine therapy	HR‐positive, HER2‐negative, advanced breast cancer with visceral metastases	[28]
	MAINTAIN	2	Palliative	Ribociclib + fulvestrant/exemestane vs. placebo + fulvestrant/exemestane	HR‐positive, HER2‐negative, advanced breast cancer, after progression on antiestrogen therapy plus CDK4/6 inhibitor	[29]
	MONARCH 3	3	Palliative	Abemaciclib + NSAI vs. placebo + NSAI	HR‐positive, HER2‐negative, advanced breast cancer	[30]
	monarcHER	2	Palliative	Abemaciclib + trastuzumab + fulvestrant vs. abemaciclib + trastuzumab vs. trastuzumab + chemotherapy	HR‐positive, HER2‐positive, advanced breast cancer	[31]
	DAWNA‐1	3	Palliative	Dalpiciclib + fulvestrant vs. placebo + fulvestrant	HR‐positive, HER2‐negative, advanced breast cancer	[32]
	DAWNA‐2	3	Palliative	Dalpiciclib + letrozole/anastrozole vs. placebo + letrozole/anastrozole	HR‐positive, HER2‐negative, advanced breast cancer, as first‐line treatment	[33]
	FAKTION	2	Palliative	Fulvestrant + capivasertib vs. fulvestrant + placebo	ER‐positive, HER2‐negative, metastatic breast cancer, after relapse or progression on AI	[34]
	CAPItello‐291	3	Palliative	Fulvestrant + capivasertib vs. fulvestrant + placebo	HR‐positive, HER2‐negative, advanced breast cancer, after relapse or progression on AI	[35]
Immunotherapy	KEYNOTE‐522	3	Neoadjuvant	Pembrolizumab + chemotherapy vs. placebo + chemotherapy	Early TNBC	[36]
	IMpassion031	3	Neoadjuvant	Atezolizumab + chemotherapy vs. placebo + chemotherapy	Early TNBC	[37]
	NeoPACT	2	Neoadjuvant	Pembrolizumab + carboplatin + docetaxel	Early TNBC	[38]
		2	Neoadjuvant	Camrelizumab + chemotherapy	Early TNBC	[39]
	SYNERGY	2	Palliative	Durvalumab + paclitaxel + carboplatin + oleclumab vs. durvalumab + paclitaxel + carboplatin	Advanced/metastatic TNBC	[40]
	ICON	2b	Palliative	Chemotherapy + ipilimumab + nivolumab vs. chemotherapy	HR‐positive, metastatic breast cancer	[41]

Abbreviations: AI, aromatase inhibitor; CDK4/6, cyclin‐dependent kinase 4/6; ER, estrogen receptor; HER2, human epidermal growth factor receptor 2; HR, hormone receptor; NSAI, nonsteroidal aromatase inhibitor; OFS, ovarian function suppression; T‐DXd, trastuzumab deruxtecan; TNBC, triple‐negative breast cancer.

## 2 SYSTEMIC THERAPY FOR BREAST CANCER

### 2.1 Chemotherapy

Chemotherapy is an important part in the comprehensive treatment of breast cancer and can prolong the survival of patients. To date, chemotherapy resistance, high incidence of adverse reactions and poor tolerance are still bottlenecks that need to be solved urgently.

As a new promising treatment modality, metronomic chemotherapy is characterized by long‐term low‐dose chemotherapy administered frequently at close and regular intervals without prolonged interruptions, which can maintain long‐term and active plasma levels of the drug, resulting in favorable tolerance [42]. At the European Society for Medical Oncology (ESMO) Congress in 2022, the METEORA‐II study reported the efficacy and safety of metronomic oral vinorelbine plus cyclophosphamide and capecitabine (VEX) versus weekly paclitaxel as first‐ or second‐line treatment of estrogen receptor (ER) positive, human epidermal growth factor receptor 2 (HER2) negative metastatic breast cancer (MBC). The results showed that the VEX regimen significantly improved time to failure (TTF) (median: 8.3 vs. 5.7 months, hazard ratio (HR) = 0.61, *p* = 0.008) and progression‐free survival (PFS) (median: 11.1 vs. 6.9 months, HR = 0.67, *p* = 0.03) compared with paclitaxel, while there was no significant difference in overall survival (OS) between the two groups (HR = 0.98; 95% confidence interval (CI): 0.59–1.63) [3]. In terms of safety, more patients rated the adverse reactions of VEX regimen as ≥ grade 3 (42.9% vs. 28.6%). This study suggests that metronomic VEX should be considered as first‐line chemotherapy.

Dose‐dense chemotherapy is achieved by shortening the intertreatment interval to minimize regrowth of tumor cells, allowing more potent antitumor activity. Dose‐dense chemotherapy has made a significant contribution to the adjuvant treatment of breast cancer, but has not been recommended by some international guidelines as the optimal chemotherapy regimen for high‐risk patients. ESMO 2022 reported long‐term survival data from the GIM2 phase III study, during a median follow‐up of 15.2 years, dose‐dense chemotherapy significantly improved long‐term survival compared with standard regimens, with an absolute benefit of 9% in both disease‐free survival (DFS) and OS [4]. The final analysis of the GIM2 study confirmed that, regardless of hormone receptor status, the use of dose‐dense therapy was associated with improved efficacy compared with standard regimens. Therefore, further studies are still needed to explore the chemotherapy regimen and dose intensity.

Taxanes are one of the most important drugs for the treatment of breast cancer and are widely used in neoadjuvant and adjuvant treatment of breast cancer, as well as palliative treatment of advanced disease. Neurotoxicity is one of the most common adverse reactions to taxanes, occurring in 60%–70%, and includes sensory, autonomic and motor toxicity [43, 44]. Dose‐dependent and cumulative neuropathy has become one of the main reasons for early termination of treatment, negatively impacting patients' quality of life and clinical outcomes. A patient‐reported outcomes (PRO) study conducted at 9 medical centers in China found that of 1234 breast cancer patients enrolled, the majority of the nanoparticle albumin‐bound paclitaxel (nab‐paclitaxel) group reported numbness in the hands and feet associated with sensation conditions (81.4%), while patients in the paclitaxel (47.2%) and docetaxel (44.4%) groups mainly reported motor and autonomic symptoms. In addition, patients in the paclitaxel (HR = 0.59, *p* = 0.008) and docetaxel (HR = 0.65, *p* = 0.02) groups had a significantly lower risk of neurotoxic effects than those in the nab‐paclitaxel group [45], which may contribute to early detection and intervention of taxane‐related neurotoxicity in breast cancer patients.

### 2.2 Targeted therapy

#### 2.2.1 HER2 targeted therapy

HER2‐positive breast cancers account for about 20% of all breast cancers, are more aggressive and have a worse prognosis. The prognosis of HER2‐positive breast cancer has been significantly improved due to the development of HER‐2‐targeted drugs, including trastuzumab, pertuzumab, pyrotinib, and trastuzumab‐emtansine (T‐DM1). However, HER2‐low cancers, defined as immunohistochemistry (IHC) 1+ or 2+ and negative in situ hybridization tests, represent 45%–55% of breast cancer patients and are unlikely to benefit from conventional HER2‐targeted drugs. The growing success of a new generation of drugs, especially the promising HER2‐directed antibody‐drug conjugates (ADC), has changed the treatment landscape for patients with HER2‐low breast cancer.

##### 2.2.1.1 Neoadjuvant therapy

HR‐positive, HER2‐positive breast cancer (triple‐positive breast cancer [TPBC]) tends to respond clinically to neoadjuvant targeted therapy more often than HR‐negative, HER2‐positive breast cancer due to crosstalk between HER2 and hormone receptor (HR) signaling pathways. The MUKDEN01 single‐arm phase II trial evaluated oral, chemotherapy‐free neoadjuvant therapy with pyrotinib, letrozole and dalpiciclib in patients with TPBC. Among the 61 TPBC patients enrolled, the total pathologic complete response rate (tpCR) was 29.5% (18/61 patients), and the objective response rate (ORR) was as high as 88.5% [5], which was comparable to the double HER2 blockade in TPBC patients with standard chemotherapy [46, 47]. During the COVID‐19 epidemic, neoadjuvant therapy with pyrotinib, letrozole and dalpiciclib in TPBC patients not only has good efficacy and manageable side effects, but also is more convenient.

##### 2.2.1.2 Adjuvant therapy

The APHINITY phase III trial has demonstrated that the dual anti‐HER2 with trastusumab and pertusumab as adjuvant treatment significantly improved iDFS among patients with HER2‐positive early breast cancer [48]. Updated results from the APHINITY trial with a median follow‐up of 8.4 years, was presented at the 2022 ESMO congress. For the primary endpoint, the addition of pertuzumab revealed a 2.6% absolute benefit for 8‐year invasive disease‐free survival (iDFS) (88.4% vs. 85.8%, HR = 0.77; 95% CI = 0.66–0.91), especially in lymph node‐positive cohort [6]. Higher 8‐year OS (92.7% vs. 92.0%, HR = 0.83; *p* = 0.078) was observed with the pertusumab‐based regimen; however, this increase has not yet reached a statistical significance. The updated data confirmed the survival benefit of adding pertuzumab to trastuzumab plus chemotherapy in HER2‐positive early breast cancer.

T‐DM1 is an HER2‐targeted ADC comprised of trastuzumab and the cytotoxic microtubule inhibitor mertansine (DM1) by a noncleavable linker according to a drug‐to‐antibody ratio (DAR) of 3.5. The 2022 San Antonio Breast Cancer Symposium (SABCS) reported the updated 5‐year survival results of the ATEMPT trial, which was designed to compare the efficacy of adjuvant T‐DM1 versus paclitaxel plus trastuzumab (TH) for stage I HER2‐positive breast cancer. Among the 497 patients, the 5‐year iDFS for T‐DM1 was 97.8%, higher than 91.3% in TH group, while the 5‐year OS for T‐DM1 and TH groups were 97.8% and 97.9%, respectively [7]. This study indicated that T‐DM1 monotherapy was effective and well‐tolerated in patients with stage I HER2‐positive breast cancer.

##### 2.2.1.3 HER2 positive MBC

Pyrotinib, an irreversible oral pan‐HER tyrosine kinase inhibitor (TKI) plus capecitabine, has shown promising efficacy as a second‐line therapy for HER2‐positive MBC [49]. The PHILA phase III trial reported the efficacy and safety of the dual anti‐HER2 regimen of pyrotinib plus trastuzumab and docetaxel (THPy) versus placebo plus trastuzumab and docetaxel in untreated HER2‐positive MBC [8]. In terms of safety, THPy was well tolerated, with diarrhea clinically manageable and no new identifiable safety signals. Notably, the median PFS assessed by an independent review of the THPy regimen has reached 33.0 months, the longest median PFS reported in the first‐line treatment of HER2‐positive MBC. The results represented in this study are expected to change the landscapes of first‐line treatment of HER2‐positive MBC.

Trastuzumab deruxtecan (T‐DXd) is a novel HER2‐targeting ADC consisting of an anti‐HER2 antibody, a cleavable tetrapeptide‐based linker and a cytotoxic topoisomerase I inhibitor (exatecan derivative, also known as DXd) [50]. At 2022 SABCS, the DESTINY‑Breast 02 study compared the efficacy of T‐DXd with physician's choice treatment in patients with HER2‐positive MBC previously treated with T‐DM1. In this study, T‐DXd significantly improved PFS (median: 17.8 vs. 6.9 months, HR = 0.36, *p* < 0.0001) and OS (median: 39.2 vs. 26.5 months, HR = 0.66, *p* = 0.0021) compared with physician's choice treatment for patients with HER2‐positive MBC before T‐DM1 [9], with no new safety signals observed. In addition, the updated OS data of DESTINY‑Breast 03 was presented at 2022 SABCS. Compared with T‐DM1, T‐DXd showed significantly improvement in OS (HR = 0.64, *p* = 0.0037) in patients with HER2‐positive MBC [10]. This updated results demonstrated remarkable survival benefit and tolerable safety for T‐DXd, supporting the T‐DXd as the second‐line standard treatment in patients with HER2‐positive MBC.

##### 2.2.1.4 HER2‐low MBC

As a new generation ADC, T‐DXd has a high DAR and cytotoxic bystander effect, allowing to target HER2‐heterogeneous tumors. A phase Ib study (DS8201‐A‐J101) initially demonstrated good antitumor activity and good tolerability of T‐DXd in patients with advanced HER2‐low breast cancer [51]. This study was followed by the DESTINY‐Breast 04 phase III randomized study comparing T‐DXd with the investigator's choice of chemotherapy in HER2‐low breast cancer, which recently met its primary endpoints. This first phase III trial found that T‐DXd significantly improved PFS (9.9 vs. 5.1 months, HR = 0.50, 95% CI: 0.40–0.63, *p* < 0.001) and OS (23.9 vs. 17.5 months, HR = 0.64, 95% CI: 0.48–0.86, *p* = 0.0028) compared with chemotherapy in HER2‐low breast cancer patients [11]. In terms of adverse reactions, the incidence of grade ≥3 adverse events in the T‐DXd group was significantly lower than that in the chemotherapy group (52.6% vs. 67.4%). Notably, the incidence of interstitial lung disease (ILD) in the T‐DXd group was 12.1%, but most cases (10.0%) were grade 1/2 ILD. Based on the promising results of DB‐04, the National Comprehensive Cancer Network (NCCN) guidelines [52] recommend T‐DXd for HER2‐low MBC patients who have received at least one chemotherapy (HR‐positive patients should be resistant to endocrine therapy).

##### 2.2.1.5 Breast cancer brain metastases

Approximately 30%–50% of patients with HER2‐positive MBC develop brain metastases. Breast cancer with brain metastases has a poor prognosis and represents the greatest clinical challenge in the treatment of HER2‐positive breast cancer. In the DESTINY‐Breast 01 study, which included 24 patients with stable brain metastases, the ORR in the T‐DXd group was 58.3%, and the median PFS was 18.1 months [12]. The Phase II TUXEDO‐1 study investigated the efficacy and safety of T‐DXd in patients with active brain metastases. Among the 15 patients with brain metastases from HER2‐positive breast cancer, the T‐DXd intracranial response rate was 73.3% (11/15 cases), the clinical benefit rate (CBR) was 86.7% (13/15 cases), and the median PFS was 14 months [13]. No new safety issues were observed during treatment, and overall quality of life as well as cognitive function was maintained [53], thus demonstrating the favorable efficacy of T‐DXd in patients with active brain metastases. Moreover, the DAISY study reported the efficacy of T‐DXd in patients with HER2‐low MBC and brain metastases. A total of 24 patients were divided into 3 cohorts, including cohort 1 (HER2 overexpressing: 12 patients), cohort 2 (HER2‐low: 10 patients) and cohort 3 (HER2‐IHC0: 2 patients), with best objective response rates of 91.7%, 30%, and 50%, respectively, and a median PFS of 13 months, 4.1 months and was not reached [14]. Overall, T‐DXd showed good antitumor activity in patients with brain metastases, which deserves further study.

Previous studies have evaluated the efficacy of TKIs in the treatment of brain metastases [54, 55]. Tucatinib is a highly selective HER2‐targeting TKI. The HER2CLIMB study demonstrated the efficacy of tucatinib in the treatment of brain metastases from HER2‐positive breast cancer [15]. Results of the final overall survival analysis showed that tucatinib plus trastuzumab and capecitabine significantly prolonged OS, both in all 291 patients with brain metastases (21.6 vs. 12.5 months) and in 174 patients with active brain metastases (21.4 vs. 11.8 months) [56], which provides convincing evidence for the use of TKIs in patients with HER2‐positive breast cancer and brain metastases.

#### 2.2.2 HER3 targeted therapy

HER3, a member of the HER receptor tyrosine kinase family, is closely related to tumor growth, metastasis, and chemoradiotherapy resistance [57]. HER3 has been reported to be overexpressed in 30% of primary breast cancers and up to 60% of metastatic tumors [58]. Patritumab‐deruxtecan (HER3‐DXd) is a HER3‐directed ADC consisting of an anti‐HER3 monoclonal antibody covalently linked to a topoisomerase 1 inhibitor payload, which is an exatecan derivative (DXd). A phase I/II study of HER3‐DXd demonstrated safety and preliminary clinical activity in HER3‐expressing MBC patients. In HR‐positive/HER2‐negative, triple‐negative breast cancer (TNBC) and HER2‐positive MBC patients, the ORR of HER3‐DXd was 30.1%, 22.6%, and 42.9%, respectively, and the median PFS was 7.4 months, 5.5 months and 11.0 months, respectively, and the median OS was 14.6 months, 14.6 months and 19.5 months, respectively [16]. The most common adverse reactions were gastrointestinal toxicity and hematologic toxicity. Moreover, 12 patients (6.6%) experienced treatment‐related ILD, the majority (4.3%) of which were grade 1 or 2, thus demonstrating an adequate safety and tolerability profile. Future studies with different subtypes with larger sample sizes are needed to confirm these findings.

#### 2.2.3 Trop‐2 targeted therapy

Trophoblast cell‐surface Ag‐2 (Trop‐2) is a transmembrane glycoprotein highly expressed in a variety of solid tumors, including breast cancer (78%) [59]. Sacituzumab govitecan (SG) is an anti‐Trop‐2 ADC consisting of a humanized monoclonal antibody (hRS7) conjugated to SN‐38, the active metabolite of irinotecan, which functions as a topoisomerase I inhibitor via a hydrolyzable linker [60]. In April 2020, SG received FDA approval for metastatic TNBC based on the results of the ASCENT trial. The TROPiCS‐02 phase Ⅲ trial aimed to investigate the efficacy of SG in patients with HR‐positive, HER2‐negative MBC who previously received endocrine therapy, cyclin‐dependent kinase 4/6 (CDK4/6) inhibitors and 2–4 lines of chemotherapy. SG significantly improved PFS (median: 5.5 vs. 4.0 months), OS (median: 14.4 vs. 11.2 months) and overall health‐related quality of life compared with physician's choice of chemotherapy [17]. The safety profile of SG was consistent with previous reports and no new safety signals were identified. Based on these results, the NCCN guidelines recommended SG for the treatment of patients with HR‐positive breast cancer who have received endocrine therapy, CDK4/6 inhibitors and at least two lines of chemotherapy.

### 2.3 Endocrine therapy

Endocrine therapy is an important treatment strategy for breast cancer, mainly applicable to patients with HR‐positive breast cancer, including neoadjuvant and adjuvant endocrine therapy for early breast cancer and palliative endocrine therapy for advanced breast cancer. Patients requiring long‐term adjuvant or maintenance therapy are more suitable for endocrine therapy.

#### 2.3.1 Neoadjuvant endocrine therapy

Neoadjuvant endocrine therapy is gaining attention as a potential alternative for the treatment of hormone‐dependent breast cancer. Giredestrant (GDC‐9545) is a new oral selective estrogen receptor degrader (SERD), which can effectively bind to the ER, block the transmission of ER signaling pathway, and correspondingly inhibit tumor growth [61]. The CoopERA BC study compared the efficacy and safety of neoadjuvant giredestrant versus anastrozole plus palbociclib in the treatment of ER‐positive, HER2‐negative, postmenopausal early breast cancer. The two groups of patients first received 2 weeks of single drug endocrine therapy. The giredestrant group showed better Ki67 inhibition (80.0% vs. 67.0%) and complete cell cycle arrest (CCCA, 25.0% vs. 5.1%) [62]. Two weeks later, posttreatment was combined with palbociclib. In the final analysis, a greater inhibition of Ki67 was observed with giredestrant at surgery (81.0% vs. 74.0%). Likewise, the giredestrant group achieved a greater CCCA during surgery (20.0% vs. 14.0%). Furthermore, ORR and pCR were similar in both groups [18]. Analysis of biomarker subgroups showed that after 2 weeks of treatment, giredestrant treatment also resulted in a significant reduction in Ki67 in progesterone receptor (PgR) negative tumors. ER and PgR protein levels were also significantly decreased after 2 weeks of treatment with giredestrant [63]. As a representative of oral SERD, drugs such as giredestrant are expected to overcome the problems that fulvestrant cannot be taken orally, but also have unfavorable factors (e.g., limited bioavailability and metabolic instability). This study provides a new treatment option for adjuvant therapy in patients with ER‐positive early breast cancer. However, whether this regimen can benefit the long‐term survival of patients with relatively high Ki67 requires long‐term follow‐up.

The benefit of chemotherapy in addition to endocrine therapy in premenopausal women with HR‐positive early breast cancer at intermediate risk of recurrence remains controversial. Results of the ADAPT and ADAPT cycle trials have been reported at the ESMO congress 2022. In premenopausal women with HR‐positive, HER2‐negative breast cancer, regardless of the recurrence score, ovarian function suppression (OFS) combined with tamoxifen or an aromatase inhibitor (AI) improves response to preoperative endocrine therapy compared with endocrine monotherapy. In addition, the remission rate of OFS plus AI was higher than that of tamoxifen, especially in younger patients ≤40‐year‐old. In the ADAPT cycle study, the response rate of endocrine therapy to OFS plus AI was comparable to that of AI monotherapy in postmenopausal patients (76.9% vs. 77.9%). Endocrine therapy regimen, recurrence score and ER and/or PgR expression were predictors of the response to preoperative short‐term endocrine therapy [19].

#### 2.3.2 Adjuvant endocrine therapy

Adjuvant endocrine therapy is an important aspect of early breast cancer treatment. Intensive adjuvant endocrine therapy can further reduce the risk of recurrence and prolong DFS and OS in breast cancer patients. For premenopausal patients with HR‐positive early breast cancer, OFS combined with endocrine therapy has become the preferred adjuvant therapy for high‐risk patients recommended by major domestic and foreign guidelines. Eight‐year follow‐up data from the ASTRA study has been reported at the 2022 American Society of Clinical Oncology (ASCO) Annual Meeting. The 8‐year DFS rate of the tamoxifen plus OFS group was higher than that of the tamoxifen monotherapy group (85.4% vs. 80.2%), and the absolute benefit rate of the 8‐year DFS rate was 5.2%. The subgroup analysis of patient survival outcomes showed that among patients aged 40–45 years, the 8‐year DFS rate of the tamoxifen plus OFS group was significantly higher than that of the tamoxifen monotherapy group (89.1% vs. 80.1%), and HER2‐negative patients (85.2% vs. 80.9%). Currently, regarding OS [64], there was no significant difference between the two groups. This study further verified the significant therapeutic benefit of OFS combined with endocrine therapy in postmenopausal patients with HR‐positive breast cancer after chemotherapy.

For patients with HR‐positive early breast cancer, the latest research progress shows that extending the time of adjuvant endocrine therapy may optimize the treatment response of some patients. In recent years, the strategy of extending adjuvant endocrine therapy has been gradually accepted and adopted by clinicians, but there are still many controversies about which types of patients need to extend adjuvant endocrine therapy and the optimal course of adjuvant endocrine therapy [20]. The final analysis of the DATA trial has been reported at ESMO Congress 2022. Postmenopausal women with disease‐free HR‐positive breast cancer who received 2–3 years of tamoxifen continued to receive adjuvant therapy with AI anastrozole and tamoxifen in a 1:1 ratio for 3 or 6 years. The 10‐year adaptive DFS was 69.1% in the 6‐year group and 66.0% in the 3‐year group (*p* = 0.073). Subgroup analysis showed that in the ER and PgR positive subgroups, the 10‐year adaptive DFS was higher in the 6‐year group than in the 3‐year group (70.8% vs. 64.4%, *p* = 0.008) [65]. The study does not recommend sequential treatment, extending AI beyond 5 years for all postmenopausal women with HR‐positive breast cancer. However, it can be considered for patients with node‐positive, ER‐ positive and PgR‐positive tumors.

The benefit of adjuvant chemotherapy, in addition to adjuvant endocrine therapy, in patients older than 70‐years with ER‐positive, HER2‐negative breast cancer remains controversial. The Unicancer ASTER 70s trial enrolled ER‐positive and HER2‐negative breast cancer patients aged ≥70 years. They evaluated the Tumor Genome Grade Index (GGI) score of all patients and compared the prognosis of patients with high GGI using endocrine therapy combined with chemotherapy versus endocrine therapy alone. In the intention‐to‐treat (ITT) population, the 4‐year OS rates were 90.6% and 89.4% in the endocrine therapy plus chemotherapy group and endocrine therapy group, respectively. OS was not significantly different between the two groups. Moreover, secondary endpoints, including breast cancer‐specific survival, invasive DFS and event‐free survival (EFS), also showed no significant difference between the two groups [21]. The results of this large phase III clinical trial showed that the addition of chemotherapy did not significantly improve OS compared with endocrine therapy alone in patients with ER‐positive, HER2‐negative, high‐GGI breast cancer.

One of the biggest concerns of young women with breast cancer is whether and when they can get pregnant. At 2022 SABCS 2022, POSITIVE trial reported that early stage HR‐positive breast cancer patients could interrupt endocrine therapy for 2 years after completing endocrine therapy for 18 months or more (no more than 30 months) to complete pregnancy, childbirth and breastfeeding without increasing the risk of recurrence. The trial recruited premenopausal HR‐positive breast cancer patients who received endocrine therapy for 18 to 30 months. Then they stopped endocrine therapy, went through a 3‐month washout period, became pregnant, childbirth and breastfeeding, and resumed endocrine therapy. Results showed that suspension of endocrine therapy had no effect on breast cancer recurrence and metastasis. The cumulative incidence of breast cancer‐free interval events was 8.9%, with no significant difference compared with the historical control (SOFT and TEXT trials) (HR = 0.81, 95% CI: 0.57–1.15). The cumulative incidence of distant relapse‐free interval events was 4.5%, with no significant difference compared with the historical control (SOFT and TEXT trials) (HR = 0.70, 95% CI: 0.44–1.12). This trial offers hope for early stage HR‐positive breast cancer patients who require childbearing to complete childbearing while maintaining good health. The long‐term prognosis of the disease requires further long‐term follow‐up [22].

#### 2.3.3 Palliative endocrine therapy

##### 2.3.3.1 Selective estrogen receptor modulators and degraders

Approximately 30%–40% of breast cancer patients treated with AI may develop gene mutations. Among them, the ESR1 gene is the most mutated gene. Mutations in ESR1 may lead to aberrant estrogen‐independent ER activation, which is a major cause of AI resistance. Lasofoxifene is a third‐generation oral selective estrogen receptor modulator. The ELAINE 1 study explored whether lasofoxifene has better antitumor activity than fulvestrant in advanced breast cancer patients with ESR1 mutation after progression on CDK4/6 inhibitors combined with AI therapy. The results showed that the median PFS was 4.04 months for fulvestrant and 6.04 months for lasofoxifene (*p* = 0.138). Moreover, ORR and CBR were not significantly different (*p* = 0.12) [23]. The efficacy of lasofoxifene needs to be explored on a larger scale.

As a kind of SERD, fulvestrant is an important choice for endocrine therapy of breast cancer. However, because the type of injection may limit the use of such therapies, oral SERD, including giredestrant, have been developed. A phase Ib study suggested that giredestrant showed good efficacy in advanced ER‐positive, HER2‐negative breast cancer [66]. The phase II acelERA BC trial enrolled patients with advanced ER‐positive, HER2‐negative breast cancer who had progressed after 1–2 lines of systemic therapy. Patients were randomly assigned to receive either giredestrant or physician's choice of endocrine monotherapy (fluoxetine or AI). The median PFS in the two groups was 5.6 months and 5.4 months, respectively (*p* = 0.18). CBR (31.8% vs. 21.1%) and ORR (12.6% vs. 7.2%) were higher in the giredestrant group than in the physician's choice group. Notably, the PFS benefit was more pronounced in patients with ESR1 mutations (median: 5.3 vs. 3.5 months, *p* = 0.061) [24]. Therefore, giredestrant showed a promising survival benefit in ESR1 mutations, but needs to be validated by larger studies.

Elacestrant is a novel oral SERD. In patients with ER‐positive, HER2‐negative MBC following progression on prior endocrine and CDK4/6 inhibitor therapy, the EMERALD trial demonstrated significantly prolonged PFS for elacestrant versus standard of care endocrine therapy (SoC). Benefits were observed in all patients and in patients with ESR1 mutant MBC [67]. In this trial, patients were randomized 1:1 to elacestrant or SoC (investigator's choice of aromatase inhibitor or fulvestrant). At SABCS in 2022, researchers reported the impact of duration of prior CDK4/6 inhibitors on PFS. The duration of prior CDK4/6 inhibitor in the metastatic setting was positively associated with PFS, the longer the duration of prior CDK4/6 inhibitor in the metastatic setting, the longer the PFS on elacestrant versus SoC. For patients that had been on a CDK4/6 inhibitor for at least 12 months, the median PFS was 3.78 months in the elacestrant group, compared with 1.91 months in the SoC group (HR = 0.613, 95% CI: 0.453–0.828). For patients with ESR1 mutations that had been on a CDK4/6 inhibitor for at least 12 months, the median PFS was 8.61 months in the elacestrant group and 1.91 months in the SoC group, with a 59% lower risk of progression or death (HR = 0.410, 95% CI: 0.262–0.634). Elacestrant was well tolerated with significantly longer PFS versus SoC, highlighting its potential role as a therapeutic option for patients with ER‐positive, HER2‐negative MBC [25].

##### 2.3.3.2 Combination with CDK4/6 inhibitors

CDK4/6 inhibitors combined with endocrine therapy have become the standard treatment strategy for HR‐positive, HER2‐negative advanced breast cancer. Currently, studies such as PALOMA‐2, MONALEESA‐2, MONARCH 3, and MONALEESA‐7 have confirmed that CDK4/6 inhibitors combined with endocrine therapy can significantly reduce the risk of disease progression compared with endocrine therapy alone [68].

Palbociclib is the first CDK4/6 inhibitor approved for HR‐positive, HER2‐negative advanced breast cancer. The PALOMA‐2 trial showed that palbociclib plus letrozole can significantly improve PFS compared with placebo plus letrozole in the first‐line treatment of ER‐positive, HER2‐negative advanced breast cancer (median: 24.8 vs. 14.5 months) [69]. The 2022 ASCO Annual Meeting reported the results of the final analysis of OS. Median OS was longer in the ITT population with palbociclib plus letrozole compared with placebo plus letrozole, but the results were not statistically significant (53.9 vs. 51.2 months, stratified one‐sided *p* = 0.3378) [26]. The PALOMA‐2 trial met its primary endpoint of improved PFS but not its secondary endpoint of improved OS. Furthermore, fulvestrant is one of the standard first‐ and second‐line endocrine therapy drugs for HR‐positive MBC. The results of the FUTURE study showed that the combination of palbociclib and fulvestrant beyond disease progression to fulvestrant monotherapy can further improve the survival of patients, achieving a median PFS benefit of 9.4 months [27].

Recent updates from the MONALEESA‐2, ‐3, and ‐7 trials demonstrated statistically significant PFS and OS benefits for ribociclib in combination with endocrine therapy in HR‐positive, HER2‐negative advanced breast cancer [70, 71, 72]. In general, patients with advanced breast cancer with visceral metastases have a poor prognosis. ESMO Congress 2022 reported pooled survival analysis of the MONALEESA‐2, ‐3, and ‐7 trials in HR‐positive, HER2‐negative breast cancer patients with visceral metastases, including those with liver metastases. For patients with visceral metastases, ribociclib combination therapy reduced the risk of disease progression by 39.0% (median: 22.1 vs. 12.7 months; HR = 0.61) and the risk of death by 19.0% (median: 49.0 vs. 46.5 months; HR = 0.81) [28]. Ribociclib combined regimen may be an effective strategy for the treatment of patients with visceral metastases. Moreover, the MAINTAIN trial evaluated fulvestrant or exemestane with or without ribociclib plus CDK4/6 inhibition in patients with HR‐positive, HER2‐negative metastatic breast cancer who had progressed on antiestrogen therapy. Compared with the placebo group, PFS was significantly improved in the fulvestrant or exemestane combined with ribociclib group (median: 5.29 vs. 2.76 months), and the 12‐months PFS rate was significantly improved (24.6% vs. 7.4%) [29]. The results showed that patients with HR‐positive, HER2‐negative MBC whose disease progressed on CDK4/6 inhibitor therapy, gained a significant PFS benefit by switching endocrine therapy and receiving ribociclib.

Abemaciclib in combination with nonsteroidal aromatase inhibitors (NSAIs) is approved as first‐line therapy for postmenopausal women with HR‐positive, HER2‐negative advanced breast cancer based on positive PFS results from the MONARCH 3 trial. The results of the second interim analysis of OS were reported at ESMO Congress 2022. The median OS in the ITT population was 67.1 months for abemaciclib plus NSAI and 54.5 months for placebo plus NSAI (*p* = 0.0301). Furthermore, the absolute benefit in OS amounted to 12.6 months. However, statistical significance has not yet been reached (preset *p *≤ 0.018). Final OS data are expected to be available in the next year [30]. The previous monarcHER trial demonstrated that trastuzumab plus abemaciclib plus fulvestrant significantly improved PFS in patients with HR‐positive, HER2‐positive advanced breast cancer compared with trastuzumab plus chemotherapy [73]. Updated data showed that the median OS of the trastuzumab plus abemaciclib plus fulvestrant was nearly 1 year longer than that of trastuzumab combined with chemotherapy (20.7 vs. 31.1 months) [31].

Dalpiciclib is the first self‐developed new highly selective CDK4/6 inhibitor in China. The DAWNA‐1 trial explored the efficacy and safety of dalpiciclib plus fulvestrant in patients with HR‐positive, HER2‐negative advanced breast cancer whose disease had progressed after prior endocrine therapy. Based on the efficacy of the interim analysis [74], it has been approved by the National Medical Products Administration in 2021. The latest follow‐up data were reported at ESMO Congress 2022. Median PFS was 16.6 months for dalpiciclib plus fulvestrant and 7.2 months for placebo plus fulvestrant, respectively. Compared with placebo, dalpiciclib reduced the risk of disease progression or death by 50% (one‐sided *p* < 0.0001) [32]. The results support dalpiciclib plus fulvestrant as a new option for patients who have progressed on prior endocrine therapy. Furthermore, the DAWNA‐2 trial evaluated the efficacy and safety of dalpiciclib plus letrozole or anastrozole in the first‐line treatment of HR‐positive, HER2‐negative advanced breast cancer. Median PFS was 30.6 months in the dalpiciclib plus letrozole/anastrozole group, which was significantly longer than placebo plus letrozole/anastrozole group by 12.4 months; in addition, the risk of disease progression or death was reduced by 49.0% [33]. The results further support this regimen as a new option for first‐line treatment.

##### 2.3.3.3 Combination with PI3K/AKT/mTOR inhibitors

The phosphoinositide 3‐kinase (PI3K)/protein kinase B (AKT)/mammalian target of rapamycin (mTOR) (PI3K/AKT/mTOR) signaling pathway is the most commonly mutated pathway in HR‐positive breast cancer, and abnormal activation of this pathway is highly correlated with resistance to endocrine therapy [75].

Capivasertib is the first highly selective oral small molecule AKT inhibitor that selectively inhibits three AKT isoforms (AKT1/2/3). The FAKTION trial explored the efficacy and safety of fulvestrant combined with capivasertib in patients with ER‐positive MBC who had progressed on AI threrapy [76]. The 2022 ASCO Annual Meeting reported that fulvestrant combined with capivasertib can significantly improve OS and PFS. The median OS of the fulvestrant plus capivasertib group and the fulvestrant plus placebo group were 29.3 months and 23.4 months, respectively (HR = 0.66, *p* = 0.035), and the median PFS were 10.3 months and 4.8 months, respectively (HR = 0.56, *p* = 0.002). In addition, the researchers found through next‐generation sequencing‐based biomarker analysis that patients with altered PIK3CA/AKT/PTEN signaling pathways benefited more from fulvestrant combioned with capivasertib [34].

CAPItello‐291 trial investigated the efficacy and safety of capivasertib combined with fulvestrant in patients with AI‐resistant HR‐positive, HER2‐negative advanced breast cancer. Patients were randomized 1:1 to receive fulvestrant with either placebo or capivasertib. 2022 SABCS reported PFS results. The median PFS was 7.2 months in capivasertib group and 3.6 months in placebo group (HR = 0.60, *p* < 0.001). In patients with AKT pathway‐altered tumors, median PFS was 7.3 months in the capivasertib group and 3.1 months in the placebo group (HR = 0.50, *p* < 0.001). Capivasertib combined with fulvestrant significantly improved PFS compared to fulvestrant alone in the overall population, and in patients with AKT pathway‐altered tumors, and may become a future treatment option in this setting [35].

### 2.4 Immunotherapy

In recent years, immunotherapy represented by immune checkpoint inhibitors has continuously made major breakthroughs in the treatment of breast cancer. Numerous clinical trials have confirmed that breast cancer patients treated with programmed death protein‐1 (PD‐1)/programmed death ligand‐1 (PD‐L1) inhibitors have shown significant survival benefits.

#### 2.4.1 Neoadjuvant immunotherapy

The KEYNOTE‐522 trial explored the addition of pembrolizumab to neoadjuvant chemotherapy in early‐stage TNBC. Preliminary results showed that pembrolizumab significantly improved pCR and EFS [77]. This study further explored the relationship between residual cancer burden (RCB) and EFS. RCB can be used to assess the amount of residual tumors in breast cancer patients after neoadjuvant therapy. RCB‐0, ‐1, ‐2, and ‐3, correspond to growing residual tumor. According to the 2022 ASCO Annual Meeting, in the pembrolizumab plus chemotherapy group and the placebo plus chemotherapy group, the proportions of EFS events in the RCB‐0, ‐1, ‐2, and ‐3 subgroups were 5.2% versus 7.3%, 17.4% versus 20.0%，25.5% versus 44.3%，72.5% versus 69.2%, respectively [36]. Elevated RCB grades were associated with poor prognosis in EFS. The addition of pembrolizumab reduced the incidence of EFS events. These results suggest that the benefit of EFS can be extended to patients who do not achieve pCR. Based on the data of the KEYNOTE‐522 trial, pembrolizumab has been approved by the National Medical Products Administration to add pembrolizumab on the basis of neoadjuvant chemotherapy, postoperative adjuvant pembrolizumab for the treatment of PD‐L1 positive patients with early high‐risk TNBC (combined positive score, [CPS] ≥20).

IMpassion031 trial compared efficacy and safety of atezolizumab versus placebo combined with nab‐paclitaxel followed by doxorubicin plus cyclophosphamide as neoadjuvant treatment for early‐stage TNBC. Atezolizumab group significantly improved pCR compared with placebo group (58% vs. 41%, *p* = 0.0044) [78]. In 2022, IMpassion031 trial reported PRO data. In the neoadjuvant period, both arms exhibited clinically meaningful declines of similar magnitude from baseline in physical, role function, and health‐related quality of life, and reported similar treatment side effects to bother at each visit [37]. Improved pCR from adding atezolizumab to neoadjuvant chemotherapy for early‐stage TNBC occurred without imposing additional treatment burden on patients.

The NeoPACT trial was designed to evaluate pembrolizumab and carboplatin plus docetaxel as neoadjuvant regimens in patients with TNBC. The 2022 ASCO Annual Meeting reported that the pCR and RCB 0 + 1 rates reached 58.0% and 69.0%, respectively. Moreover, the 2‐year EFS was 89.0% [38]. The results of this study provide a new option for the neoadjuvant treatment of TNBC.

Camrelizumab is the third self‐developed PD‐1 monoclonal antibody approved in China, and its combination with chemotherapy has achieved good efficacy in the treatment of advanced TNBC [79]. The 2022 ESMO Congress reported the efficacy and safety of camrelizumab combined with chemotherapy as neoadjuvant therapy for patients with early‐stage TNBC. The patient received four cycles of camrelizumab plus nab‐paclitaxel and four cycles of camrelizumab plus epirubicin plus cyclophosphamide. In 20 patients with an evaluable response, the total pCR was 65.0% and the ORR was 95.0% [39]. In patients with early‐stage TNBC, neoadjuvant therapy with camrelizumab plus nab‐paclitaxel and anthracycline‐based chemotherapy showed high pCR rates and an acceptable safety profile. Further studies are needed to validate these findings.

#### 2.4.2 Palliative immunotherapy

With the advent of immunotherapy, patients with advanced TNBC have gained more survival benefits. The SYNERGY trial explored the efficacy and safety of durvalumab, paclitaxel and carboplatin with or without the anti‐CD73 antibody oleclumab in a first‐line chemoimmunotherapy trial in patients with advanced TNBC. Oleclumab is a monoclonal antibody against CD73, which is responsible for the production of immunosuppressive adenosine in the tumor microenvironment. A combination of a PD‐1/PDL‐1 immune checkpoint inhibitor and an adenosine‐targeted inhibitor designed to enhance the immune response. According to the 2022 ESMO Congress, the CBRs of the combined oleclumab group and the noncombined group were 43.0% and 44.0%, respectively [40]. The addition of oleclumab did not improve response rates to the combination of chemotherapy and immunotherapy.

Studies of immune checkpoint inhibitors in HR‐positive advanced breast cancer, which is considered an immunologically “cold” tumor, have rarely been reported. The ICON trial explored chemotherapy in combination with the PD‐1 monoclonal antibody nivolumab and the CTLA‐4 monoclonal antibody ipilimumab. Median PFS was 5.1 months in the combination group and 3.7 months in the chemotherapy group (HR = 0.94), respectively. Moreover, the median OS was 20.9 months versus 19.9 months (HR = 1.13) [41]. The addition of nivolumab and ipilimumab to chemotherapy did not show a clear benefit, and patients receiving immunotherapy experienced more immune‐related adverse events. Further biomarker analysis will be important to determine whether subgroup studies are needed.

## 3 CONCLUSIONS AND FUTURE OUTLOOK

Breast cancer is the most serious malignant tumor that threatens women's health. With the development of systemic therapy for breast cancer, the survival time of patients has been significantly prolonged. In 2022, many breakthroughs have been made in the field of breast cancer clinical and translational research, and new treatment concepts will emerge in an endless stream.

In the era of precision medicine, chemotherapy remains the cornerstone of breast cancer treatment. Chemotherapy based on drugs such as anthracyclines and taxanes has significantly improved the survival rate of breast cancer patients. Researchers are still working on finding new treatments. For high‐risk early‐stage breast cancer, intensive doses can improve the efficacy of chemotherapy. Metronomic chemotherapy has shown therapeutic advantages in advanced breast cancer with its single low dose, good tolerance and multiple antitumor mechanisms. At present, traditional chemotherapy is challenged by various targeted drugs such as ADCs. Clinical trials based on molecular subtypes and gene expression profiles of breast cancer may become a hotspot for future research.

In terms of targeted therapy, new ADCs such as T‐DXd not only combine the selectivity of targeted therapy and the cytotoxicity of chemotherapy, but also have a “bystander killing effect,” and have achieved remarkable results in the treatment of HER2‐positive and HER2‐low breast cancer. As the first phase III trial to report positive HER2‐low MBC results, the DESTINY‐Breast 04 study changes the treatment landscape for patients with HER2‐low breast cancer, raising the possibility of improving the clinical interpretation of HER2 status from the current dualistic pattern to the three subtypes. More studies are needed to confirm the encouraging results of novel ADCs and to further define the cutoff of HER2 expression at which anti‐HER2 therapies are effective. The success of the new dual‐target (pyrotinib combined with trastuzumab) combined with chemotherapy provides a new option for the first‐line treatment of HER2‐positive advanced breast cancer. In the future, combination therapy of multiple targeted drugs with different mechanisms of action will become the mainstream treatment strategy for HER2‐positive breast cancer. Furthermore, fully oral, chemotherapy‐free combination neoadjuvant therapy with pyrotinib, letrozole and dalpiciclib provides another option for TPBC patients. Head‐to‐head trials are needed to confirm the encouraging results of chemotherapy‐free therapy in TPBC.

For endocrine therapy, the appropriate drug and the mode of administration for neoadjuvant endocrine therapy needs to be determined. Further studies are warranted to accurately screen sensitive populations for endocrine therapy and overcome endocrine resistance. In particular, the optimal therapeutic strategy following resistance to CDK4/6 inhibitors and the sequential deployment of these endocrine drugs for favorable outcomes remains to be explored.

Additionally, immunotherapy can significantly prolong survival and improve the quality of life of TNBC. However, the identification of immunotherapy‐sensitive populations, the selection of combination regimens and biomarkers for predicting efficacy still need further exploration. Currently, studies on the efficacy of immunotherapy on TNBC are underway, and more studies are expected to provide alternatives and evidence‐based recommendations for immunotherapy of TNBC in the future.

At present, innovative drugs for breast cancer are constantly being developed, and clinical trials are emerging one after another. Interdisciplinary and multiomics data fusion can bring new concepts to breast cancer research and bring double benefits of prognosis and quality of life to breast cancer patients.

## AUTHOR CONTRIBUTIONS

**Jingtong Zhai**: Writing – original draft (equal); writing – review & editing (equal). **Yun Wu**: Writing – original draft (equal); writing – review & editing (equal). **Fei Ma**: Conceptualization (lead); supervision (lead). **Virginia Kaklamani**: Writing – review & editing (equal). **Binghe Xu**: Conceptualization (lead); supervision (lead).

## CONFLICT OF INTEREST STATEMENT

Professor Binghe Xu and Fei Ma are members of the *Cancer Innovation* Editorial Board. To minimize bias, they were excluded from all editorial decision‐making related to the acceptance of this article for publication. The remaining authors declare no conflicts of interest.

## ETHICS STATEMENT

Not applicable.

## INFORMED CONSENT

Not applicable.

## Data Availability

Data sharing is not applicable to this article, as no data sets were generated or analyzed during the current study.
